# Pharmacokinetics and Biodistribution of Zinc-Enriched Yeast in Rats

**DOI:** 10.1155/2014/217142

**Published:** 2014-08-17

**Authors:** Shuangqing Zhang, Yan Zhang, Ning Peng, Haibo Zhang, Juan Yao, Zhihong Li, Liegang Liu

**Affiliations:** ^1^Division of Nutrition and Health, Angel Yeast Co. Ltd., 168 Chengdong Avenue, Yichang 443003, China; ^2^Department of Nutrition and Food Hygiene, Hubei Key Laboratory of Food Nutrition and Safety, State Key Laboratory of Environment Health (Incubation), School of Public Health, Tongji Medical College, Huazhong University of Science and Technology, Wuhan 430030, China

## Abstract

Zinc-enriched yeast (ZnY) and zinc sulfate (ZnSO_4_) are considered zinc (Zn) supplements currently available. The purpose of the investigation was to compare and evaluate pharmacokinetics and biodistribution of ZnY and ZnSO_4_ in rats. ZnY or ZnSO_4_ were orally administered to rats at a single dose of 4 mg Zn/kg and Zn levels in plasma and various tissues were determined using inductively coupled plasma-optical emission spectrometry. Maximum plasma concentration values were 3.87 and 2.81 *μ*g/mL for ZnY and ZnSO_4_, respectively. Both ZnY and ZnSO_4_ were slowly eliminated with a half-life of over 7 h and bone had the highest Zn level in all tissues. Compared to ZnSO_4_, the relative bioavailability of ZnY was 138.4%, indicating that ZnY had a significantly higher bioavailability than ZnSO_4_.

## 1. Introduction

Essential trace elements are very important for the proper functioning of living organisms, such as growth and maintenance, neuromodulation, and regulation of cellular function, and thus their deficiency is associated with an enormous health risk that can ultimately lead to death [1]. Zinc (Zn) is an essential trace element for plants, microorganisms, animals, and humans and plays numerous central roles in structure and function of proteins, metabolism of RNA and DNA, signal transduction, gene expression, and regulation of apoptosis [2]. Zn deficiency affects nearly two billion people in the developing countries and is associated with many diseases, including depressed growth, diarrhea, impotence and delayed sexual maturation, alopecia, eye and skin lesions, impaired appetite, altered cognition, impaired host defense properties, defects in carbohydrate utilization, and reproductive teratogenesis [3, 4]. Zn deficiency is usually caused by insufficient dietary intake but can be associated with many diseases, such as malabsorption, acrodermatitis enteropathica, chronic liver disease, chronic renal disease, sickle cell disease, diabetes, malignancy, and other chronic illnesses [4]. Zn supplements help prevent disease and reduce mortality, especially among children with low birth weight or stunted growth. Zn supplements include Zn chloride and Zn sulfate (ZnSO_4_) as inorganic salts for parenteral administration, Zn gluconate, Zn gluconate/glycine, Zn acetate, and Zn propionate as salts of organic acids for oral usage, and Zn-enriched yeast (ZnY) or Zn-enriched grains as biologically organic forms for oral intake [5].

ZnY, a biological source of Zn with rich proteins, peptides, and amino acids, is naturally integrated by the growing yeast into its own structure to improve the bioavailability of Zn and reduce the side effects of Zn. ZnY is used as the raw material of foods, functional foods, and medicines, such as dairy products, biscuits, beverages, and flour. So far, only two studies of ZnY reported that ZnY was more bioavailable than Zn gluconate in rat liver and human [6, 7]. The purpose of the present investigation was to compare and evaluate pharmacokinetics and biodistribution of ZnY and ZnSO_4_ in rats.

## 2. Materials and Methods

### 2.1. Materials

ZnY was provided by Angel Yeast Co. Ltd. (Yichang, Hubei, China). ZnSO_4_ was obtained from Sigma Aldrich (St. Louis, MO, USA). Nitric acid and perchloric acid were the guaranteed reagents. All other reagents were of analytical grade and obtained through commercial sources.

### 2.2. Pharmacokinetics and Biodistribution

All procedures involving animals were approved by our Institutional Animal Care and Use Committee. Adult Sprague-Dawley rats, weighing 250 ± 11 g, were obtained from Jackson Laboratory (Bar Harbor, ME, USA) and housed in standard cages and allowed free movement and access to food and water during the whole experiment. The rats were randomly assigned into two groups and orally given either ZnY or ZnSO_4_ at a single dose of 4 mg Zn/kg. At designated time-points, blood was collected in a heparin-coated tube and plasma was separated by centrifugation at 5,000 g for 10 min. Supernatant was then transferred to a clean tube and immediately stored at −80°C until analysis. Immediately after the last time-point blood collection, rats were sacrificed using carbon dioxide and tissues (liver, heart, pancreas, spleen, kidney, and bone) were immediately excised. All tissue samples washed with tridistilled water were frozen at −80°C until analysis.

### 2.3. Zn Determination

Zn levels in plasma, liver, heart, pancreas, spleen, kidney, and bone were determined using inductively coupled plasma-optical emission spectrometry (Model 720, Agilent Inc., CA, USA). Samples were prepared as follows: 100 *μ*L of plasma or weighted tissue specimen was pretreated with the mixture of nitric acid and perchloric acid (20 : 1, v/v) overnight and heated at the temperature of 180–200°C until samples were completely digested, that is, until the solutions were colorless and clear. The remaining acid solution was cooled and diluted with tridistilled water to an appropriate concentration for the assay. Quality control samples were employed for the validation of analytical method and the analytical error was less than 11%.

### 2.4. Data Analysis

Pharmacokinetic parameters of ZnY and ZnSO_4_ were calculated using noncompartmental analysis of WinNonlin software (Version 5.2.1, Pharsight Corp., Mountain View, CA, USA). Results are presented as mean ± standard deviation (SD). The comparison for difference between parameters or between groups was analyzed using one-way analysis of variance (ANOVA). Differences were considered statistically significant at *P* < 0.05.

## 3. Results and Discussion

Although food is the major source of Zn, low Zn content in diet and many adverse nutritional factors are barriers to prevention and treatment of Zn deficiency [8, 9]. Zn chloride, ZnSO_4_, and Zn oxide are often employed for food fortification; however there are serious shortcomings for those compounds. Zn chloride causes some adverse effects, ZnSO_4_ renders unpalatable flavor, and water-insoluble Zn oxide precipitates in liquid foods; therefore, those compounds are used only in low quantities and in solid foods. ZnY has high solubility and a soft taste, and it does not modify the sensorial characteristics of food.

**Figure 1:**
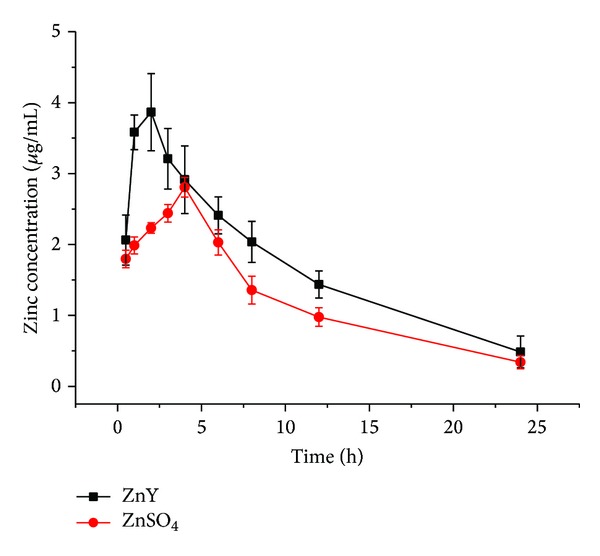
The mean plasma concentration-time profiles of ZnY and ZnSO_4_ after a single oral administration of zinc compounds at a dose level of 4 mg Zn/kg (*n* = 3).

The concentration-time profile of ZnY and ZnSO_4_ in plasma is shown in Figure 1 and their pharmacokinetic parameters are listed in Table 1. Peak plasma concentration (*C*_max⁡_) and the time to reach *C*_max⁡_ (*T*_max⁡_) were 3.87 *μ*g/mL and 2 h for ZnY and 2.81 *μ*g/mL and 4 h for ZnSO_4_, respectively. *C*_max⁡_, AUC_0–24 h_, and AUC_0–*∞*_ ratio values of ZnY to ZnSO_4_ were 137.7%, 138.4%, and 139.2%, respectively, indicating that ZnY had a significantly higher bioavailability than ZnSO_4_. Both ZnY and ZnSO_4_ were slowly eliminated* in vivo* with respective *t*_1/2,*λz*_ values of 7.68 and 7.93 h. During ZnY fermentation in the presence of Zn chloride or ZnSO_4_, a specific strain of yeast produced specific zinc compounds conjugated with proteins, peptides, and amino acids, which helped improve bioavailability of ZnY compared to inorganic Zn via enhanced intestinal absorption. Our findings with ZnY are consistent with previous studies in which animal proteins could improve Zn absorption [6, 10], which could physiologically illustrate protein as the major dietary source of Zn.

**Figure 2:**
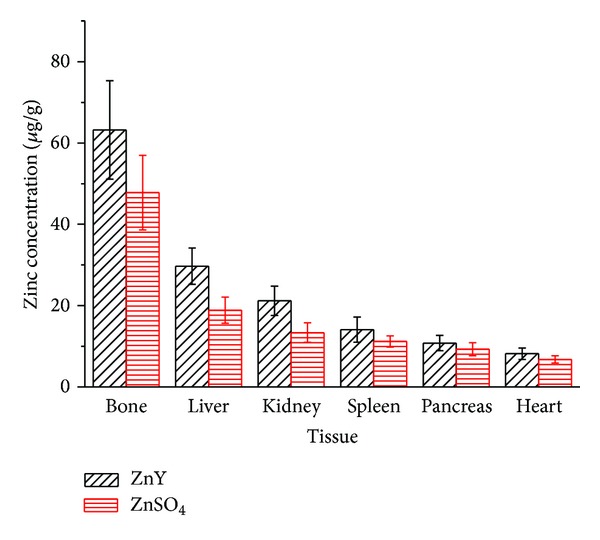
Distribution profiles of ZnY and ZnSO_4_ in rat tissues at 24 h after a single oral administration of zinc compounds at a dose level of 4 mg Zn/kg (*n* = 3).

Zn levels in liver, heart, pancreas, spleen, kidney, and bone are presented in Figure 2. The biodistribution results demonstrated that Zn was widely distributed in various rat tissues after oral administration of ZnY and ZnSO_4_ in which bone had the highest Zn level, and ZnY showed higher Zn level than ZnSO_4_ in each tissue. The results are in agreement with previous report [11]. Zn is essential for bone mineralization and bound to the mineral matrix. Bone growth retardation is usually associated with Zn deficiency [12]. Zn can stimulate proliferation, differentiation, and protein synthesis in osteoblastic cells and inhibit the formation of osteoclastic cells from bone marrow cells [13–15]; therefore Zn plays an important role in the preservation of bone mass by stimulating bone formation and inhibiting bone resorption. Despite the lower Zn levels in tissues other than bone, Zn metalloenzymes are widespread throughout body organs and play crucial roles in many physiologic processes. Other organs that had significant Zn concentration were liver, kidney, spleen, and pancreas; liver, spleen, and pancreas are closely associated with Zn metabolism while kidney is related with Zn excretion. Small intestine is the major absorption organ of Zn, and a part of absorbed Zn is stored bound to intestinal metallothionein, while the rest of Zn is transported by blood albumin and bound to hepatic metallothionein in liver or involved in a wide range of metabolic functions in many tissues [11].

## 4. Conclusions

In conclusion, Zn from ZnY was more bioavailable than zinc from inorganic zinc salts ZnSO_4_; disposition and biodistribution of Zn in ZnY were similar to those of other sources of Zn.

## Figures and Tables

**Table 1:** The pharmacokinetic parameters of ZnY and ZnSO_4_ after a single oral administration of Zn compounds at a dose level of 4 mg Zn/kg. Values are presented as mean ± SD (*n* = 3).

Parameter	Unit	ZnY	ZnSO_4_
t_1/2,*λz*_	h	7.68 ± 1.82	7.93 ± 0.87
AUC_0–24 h_	h*·μ*g/mL	40.49 ± 5.48	29.26 ± 3.01
AUC_0–*∞*_	h*·μ*g/mL	46.24 ± 8.97	33.22 ± 4.43
*V*	mL/kg	954 ± 77	1381 ± 50
Cls	mL/h/kg	88.8 ± 17.7	121.9 ± 17.0
MRT_0–24 h_	h	7.94 ± 0.56	7.84 ± 0.36
MRT_0–*∞*_	h	11.20 ± 2.31	11.09 ± 1.25

t_1/2,*λz*_: terminal half-life; AUC_0–24 h_: area under curve from 0 to 24 h; AUC_0–*∞*_: area under curve from 0 to infinite time; Cls: systemic clearance; *V*: volume of distribution; MRT_0–24 h_: mean residence time from 0 to 24 h; MRT_0–*∞*_: mean residence time from 0 to infinite time.
